# Enhancing High-Frequency Dielectric Properties of Beta-SiC Filled Nanocomposites from Synergy between Percolation and Polarization

**DOI:** 10.3390/ma11091699

**Published:** 2018-09-13

**Authors:** Cheng Peng, Yefeng Feng, Jianbing Hu

**Affiliations:** School of Materials Science and Engineering, Yangtze Normal University, Chongqing 408100, China; feng_ye_feng@126.com (Y.F.); hjb2008@163.com (J.H.)

**Keywords:** high-frequency, dielectric, nanocomposite, percolation, polarization

## Abstract

Promising comprehensive properties, including high permittivity, low dielectric loss, high breakdown strength, low electrical conductivity, and high thermal conductivity, are very hard to simultaneously obtain in high-frequency applicable polymer nanocomposite dielectrics. Instead of traditional electric percolation, in this work, a novel route based on a synergy between electric percolation and induced polarization has been raised to prepare 0–3 type nanocomposites with an enhanced high permittivity (high-k) property and low loss at high frequency. This work aimed at optimizing that synergy to achieve the favorable properties mentioned above in composite dielectrics used at high frequencies such as 1 MHz and 1 GHz. Conductive beta-SiC nanoparticles with a particle size of ~30 nm were employed as filler and both insulating poly(vinyl alcohol) and polyvinyl chloride were employed as polymer matrices to construct two composite systems. Utilizing polyvinyl chloride rather than poly(vinyl alcohol) realizes higher comprehensive electrical properties in composites, ascribed to optimization of that synergy. The optimization was achieved based on a combination of mild induced polarization and polarization-assisted electric percolation. Therefore, this work might open the way for large-scale production of high-frequency applicable composite dielectrics with competitive comprehensive electrical properties.

## 1. Introduction

High-permittivity (high-k) materials with low dielectric loss and high electric breakdown strength have become a focus and incurred much research interest in the field of high-density energy storage devices used under high frequencies [1]. It has been universally accepted that a high energy storage density for dielectric materials will be obtained based on a combination of high-k property and a high electric breakdown strength in those materials [2]. Usually, polymer materials have desirable high breakdown strength and easy processability, but they have undesirable low dielectric permittivity and poor heat tolerance [3], caused by their covalent bond structures. Contrary to polymer materials, high-k ceramic materials have a favorable high permittivity and strong thermal resistance, but they have an undesirable low breakdown strength and high mechanical brittlement [4], that stems from their ionic bond structures. Nowadays, the hybridization strategy [5,6,7] has been widely used to achieve high-performance materials. Therefore, the composite dielectric materials prepared via blending polymer and high-k ceramic have been largely developed to combine the advantages of both components [8]. In these composite dielectrics, adding high-k ceramic with a higher Young’s modulus compared to the polymer would improve the mechanical strength of the polymer and thus the electrical breakdown strength of the polymer [9,10]. Furthermore, the processability of composite dielectrics would be well retained, attributed to a low Young’s modulus (namely high flexibility) of the polymer component. Note, that the great development in varied ferroelectric materials [11,12,13,14,15] has contributed to the design and preparation of the above high-k ceramic/polymer composite dielectrics, because of the desirable ultrahigh permittivity in ferroelectric ceramics (such as barium titanate) and relatively high permittivity in ferroelectric polymers (such as polyvinylidene fluoride).

During past decades, the high-k composite dielectrics consisting of polymer and ferroelectric ceramic have achieved great success [16]. In order to obtain a desirably high permittivity in these composite dielectrics, the researchers must add a very high volume concentration of ceramic into the polymer, based on classic series and parallel effective dielectric models [17]. However, that high concentration of ceramic filler in the polymer matrix results in an inhomogeneous distribution and aggregation of ceramic filler in the entire composite. In this case, mechanical properties such as flexibility would be reduced for the composite. If that added ceramic has a nanometer size, mechanical properties of the composite would be severely damaged because of a rather large specific area and strong agglomeration of nano-sized ceramic particles. In order to depress the aggregation of the nanoparticles, the interface compatibility between nanoparticles and polymer should be improved, by tuning the nanoparticles surface chemistry (namely organic-modifying the surface of inorganic nanoparticles) [18,19,20,21]. Apart from the ferroelectric ceramics and polymer-based high-k composite dielectrics discussed above, those which are filled with electrically conducting materials have also gained huge success [22]. Usually, both the interface polarization [23] and electrical percolation [24] theories are employed to explain the abnormal high-k property obtained in those conductor/polymer composite dielectrics.

When an external electric field is exerted onto the composite, mobile charge carriers inside that composite accumulate in the conductor/polymer interface regions. The accumulation of charge carriers stems from a significant difference between the two components with respect to either permittivity or conductivity. As the electric conductance mechanism is about to transform from the non-Ohmic conduction mechanism to the Ohmic one (near the percolation threshold), the permittivity and conductivity of the entire composite are significantly improved based on a slight increase of volume concentration of the conducting filler. Aiming at avoiding a completely Ohmic conductance across the entire composite, the researchers must introduce a low volume concentration of conductor filler into the polymer matrix. In this case, a relatively high mechanical property would be retained in these composites [25]. However, these composites as dielectric materials are unable to withstand a higher applied electric field, ascribed to both the large dielectric loss and leakage conductance.

Although the desirable dielectric properties at low frequencies have been realized in plenty of organic-inorganic composite dielectric materials, discovering how to simultaneously achieve high permittivity and low dielectric loss in these composite dielectrics at high frequencies is still challenging. Meanwhile, high electric breakdown strength and thermal conductivity have been emphasized for these composite dielectrics [26]. In our prior work [27], an interface induced polarization between inorganic low band-gap Si-based ceramics and organic high polarity polymers has been found to contribute to a greatly elevated high permittivity for composites at low frequencies. For the sake of simultaneously achieving the favorable high permittivity and low loss in conductor/polymer composite dielectrics at high frequencies, in this work, we would like to present a novel and facile strategy for obtaining the desired dielectric performances in two 0–3 type nanocomposite systems based on physically blending polarity polymers with conductive beta-SiC nanoparticles.

To begin with, neat beta-SiC nanoparticles were used as filler to prepare composites owing to their low intrinsic band gap (ca. 2.20 eV) and high inborn conductivity (ca. 0.67 S·m^−1^) [28,29]. Thereby, high induced polarization and strong electric percolation would be expected to occur in these composites. Furthermore, high polarity poly(vinyl alcohol) and medium polarity polyvinyl chloride were employed as polymer matrices. Varied inherent polarity in both matrices would expect to result in varied induced polarization [30] and dielectric properties for two different composite systems (the varied polymer matrices but the same nanofiller). Ultimately, two different nanocomposite systems were fabricated into films through a facile solution cast process, and dielectric, conductive, and electric breakdown properties of both systems were thoroughly investigated. Desirable dielectric, electrical conductive, electric breakdown, and thermal conductive properties were successfully obtained in a beta-SiC filled polyvinyl chloride based nanocomposite system, due to an optimized synergy effect between filler/matrix interface induced polarization and electrical percolation of the filler. Both mild induced polarization and polarization-assisted percolation were inferred to be responsible for the achieved high permittivity, low loss, low conductivity, and high breakdown strength in a polyvinyl chloride based composite system employed at a high-frequency range. Furthermore, a favorable high thermal conductivity was gained in that system. Therefore, this work might open the way for the large-scale preparation of high-performance composite dielectrics that would be used at high frequencies (such as 1 MHz and 1 GHz).

## 2. Materials and Methods 

Silicon carbide nanoparticles belonging to the beta crystalline form (β-SiC NPs) were bought from Shanghai Xiangtian Nanomaterials Co., Ltd. (Shanghai, China). β-SiC NPs (real density, ca. 3.2 g cm^−3^) were washed with absolute ethanol three times to get rid of impurities, followed by drying at 180 °C for 6 h before use. Poly(vinyl alcohol) (PVA, 17-88, polymerization degree ca. 1700, hydrolysis percentage ca. 88 mol %, molecular weight (*M*_w_) ca. 83300, polydispersity index (PDI) 1.24) was purchased from Shanxi Sanwei Co., Ltd. (Shanghai, China). Polyvinyl chloride (PVC, 5401T, *M*_w_ ca. 93700, PDI 1.22) was obtained from Shanghai Micro-analysis Technology Co., Ltd. (Shanghai, China). Absolute ethanol (99.7%, AR grade) and tetrahydrofuran (THF, 99.5%, AR grade) from Aladdin, Shanghai, China were used as received.

Both nanocomposite systems, namely SiC/PVA and SiC/PVC nanocomposite systems, were prepared into films through a facile solution cast method [31] from a suspension with several designed volume concentrations (including 0 vol %, 0.10 vol %, 0.20 vol %, 0.30 vol %, and 0.40 vol %) of SiC NPs in high viscosity polymer solutions on neat glass slide substrates. For the SiC/PVA composites system, deionized (DI) water was used as polymer solvent and the films were formed at 50 °C. For SiC/PVC composite system, THF acted as the polymer solvent and the films were made at 25 °C. Employing high viscosity polymer solutions aimed at avoiding the sedimentation of high-density SiC NPs (compared with polymer materials) during the evaporation of solvents, resulting in a more even dispersion of SiC NPs in the polymer matrices. After a heat treatment in a vacuum oven for 4 h to remove air defects (100 °C for PVA based composites and 80 °C for PVC ones), all of the films (an average thickness, ca. 60 μm) were peeled off from glass slides, followed by sputtering with Au on both surfaces to form electrodes for following electrical properties measurements.

An X-ray diffraction (XRD) pattern was obtained using a Rigaku D/max 2400 diffractometer (Rigaku Corporation, Tokyo, Japan) with an X-ray wavelength of 1.542 Å (Cu Kα radiation, 40 kV, 100 mA), 2-theta diffraction angle at 10°–90°, rate of 15°/min, and step of 0.02°. Field-emission scanning electron microscopy (FE-SEM) image was obtained from a JSM-6700F (JEOL, Akishima, Japan) at 5 kV. A particle size distribution histogram result was obtained using a Winner2000M laser particle size analyzer (Ji’nan Weina Granule Technology Co., Ltd., Ji’nan, China). Electric breakdown strength data were obtained on an auto voltage withstanding tester (RK2674B, Shanghai Shuangxu Electronics Co., Ltd., Shanghai, China). Dielectric and alternative current (ac) conductive properties at room temperature were achieved by HP4284A (Shenzhen Jiagelun Electronic Instrument Co., Ltd., Shenzhen, China, testing frequencies varying from 100 Hz to 1 MHz) and HP4287A (Dongguan Xinfeiyu Instrument Co., Ltd., Dongguan, China, frequencies from 1 MHz to 1 GHz) LCR meters with a bias voltage of 1 V. Au electrodes were formed on both surfaces of films through a JEOL JFC-1600 auto fine coater before electrical properties measurements were taken. Thermal conductivity results were achieved by a thermal conductivity meter (YDR-905, Beijing Beixin Future Electronic Instrument Co., Ltd., Beijing, China).

## 3. Results and Discussion

### 3.1. Characterization of Used SiC NPs

**Figure 1:**
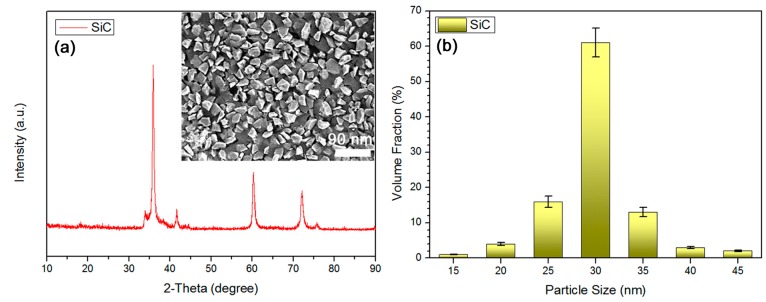
(**a**) X-ray diffraction (XRD) confirmed beta crystalline form for SiC, SEM (inset) verified high geometrical asymmetry for SiC, and (**b**) particle size distribution histogram suggested 30 nm as average particle size and a narrow particle size distribution for SiC.

The composition of applied SiC NPs was characterized by the XRD results as shown in Figure 1a. SiC NPs were proved to belong to the beta crystalline form and they possessed a structure of a cubic crystal system, based on MDI Jade 5.0 analysis software (standard card number is JCPDS 29-1129 for present 3C-SiC). The diffraction angles (2-theta) at 36°, 41.6°, 60°, 72°, and 75.8° should be assigned to crystal indices of (111), (200), (220), (311), and (222) for beta-SiC, respectively [32]. The 2-theta at 34° should be ascribed to the stacking faults formed in beta-SiC crystal [33]. The morphology of SiC NPs was studied by surface SEM results as exhibited in the inset of Figure 1a. The irregular 3D shape was observed for SiC NPs, indicating a high geometrical asymmetry for employed SiC NPs. SiC NPs were observed to have a nanoscale size. The average particle size and particle distribution of SiC NPs were obtained by the particle size distribution histogram results as displayed in Figure 1b. A relatively narrow particle size distribution (15–45 nm) was confirmed for present SiC NPs. The NPs with a particle size of 30 nm accounted for ca. 60 vol % among all the NPs with varied particle sizes, suggesting 30 nm as the average particle size (average diameter) for those used SiC NPs.

### 3.2. Dielectric, Conductive and Breakdown Properties of Nanocomposites

**Figure 2:**
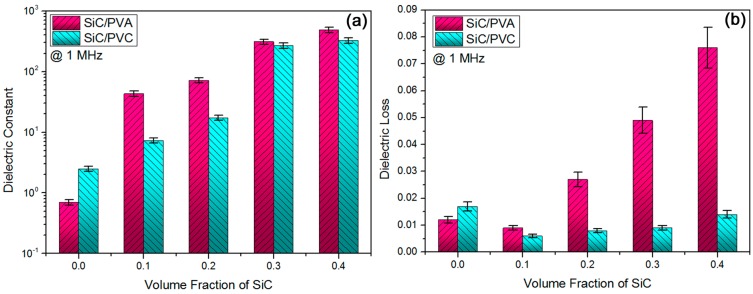
(**a**) Dielectric constant data showed both induced polarization and electric percolation in two composite systems, and (**b**) loss results suggested superiority of polyvinyl chloride (PVC)-based system to polyvinyl alcohol (PVA)-based system.

Recently, dielectrics with high permittivity, low dielectric loss, low electric conductivity, and high electric breakdown strength have attracted much investigation interest in the fields of high-frequency electronic components and devices [34]. In this work, combining the classic electric percolation with interface induced polarization, instead of single electric percolation, was carried out to optimize the electrical properties of nanocomposite dielectrics at high frequency rather than at low frequency. In Figure 2a, the dielectric constant results (at a high measuring frequency, 1 MHz) of two as-prepared composite systems as a function of SiC volume fraction were obtained. As for both composite systems, an increase of volume concentration of SiC NPs could improve the dielectric constant of composites, due to a higher electrical conductivity of beta-SiC compared to the two kinds of polymer matrices [35]. However, the dielectric constant of the SiC/PVA composite system was clearly elevated at a lower SiC volume fraction compared to that of the SiC/PVC composite system. This may originate from a higher intrinsic polarity for PVA than PVC (namely, a stronger induced polarization between SiC and PVA than that between SiC and PVC) [36]. For instance, the permittivity of PVA-based composites increased to ca. 43 from 0.7, when the SiC concentration was elevated from 0 wt % to just 10 vol %. The permittivity of PVC-based composites was enhanced to ca. 267 from 2.5, while SiC loading concentration was increased from 0 vol % to 30 vol %.

As mentioned above, apart from interface induced polarization between Si-based ceramic filler and polarity polymer matrix [37], a classic electric percolation among conductive beta-SiC NPs [38] should exist in the present two nanocomposite systems. Based on Figure 2a, a quick elevation for the ratio of composite permittivity and polymer permittivity (*ε*_composite_/*ε*_polymer_) was confirmed in two composite systems (see Graphic Abstract), while SiC volume fraction was increased from 20 vol % to 30 vol %. This suggested an electric percolation behavior of conducting SiC NPs across polymer matrices in both composite systems. It could be inferred that the critical volume fraction for realizing electric percolation of presently employed beta-SiC NPs (ca. 30 nm of the particle size) in insulating polymer matrices is very close to 30 vol %. In Figure 2a, the highest permittivity (at 1 MHz and 40 vol % of SiC concentration) was measured to be ca. 487 (*ε*_composite_/*ε*_polymer_ = 695) for a PVA-based composite system and ca. 324 (*ε*_composite_/*ε*_polymer_ = 129) for a PVC-based system. A synergy between electric percolation and induced polarization (being superior to electric percolation) is responsible for the high permittivity (high-k) feature achieved in the two composite systems, and the permittivity (over 300 at 1 MHz) of the two composites could highlight their advantages as high-frequency oriented dielectric materials.

Usually, a high permittivity of composite materials is achieved at the cost of a low dielectric loss, and simultaneously obtaining high permittivity and low loss in composite dielectrics have long been pursued [39]. In Figure 2b, the dielectric loss results corresponding to the permittivity results in Figure 2a were exhibited. For two composite systems, the loss was primarily reduced and later increased with an increase of the SiC volume concentration. The lowest loss was determined at 10 vol % of SiC loading concentration for both systems, attributed to a sharp elevation of the mechanical modulus of the materials with SiC concentrations increasing from 0 vol % to 10 vol %. An increase of the modulus resulting from the introduction of hard inorganic SiC NPs could reduce ion-leakage-induced dielectric loss in polymer matrices with a relatively low modulus [40]. However, further increasing the SiC concentration (from 10 vol % to 40 vol %) would not notably enhance the modulus of composites, and it would lead to an increase of SiC/polymer interface induced polarization [41] in addition to a higher probability of percolation. Thus, the loss was improved, once SiC was increased from 10 vol %.

With regard to the SiC/PVA composite system, the loss results of composites except for 10 vol % SiC-filled composite were found to be far higher than in pure PVA material. For example, 40 vol % SiC-filled composite had a loss (0.076 at 1 MHz) ca. six times of that in neat PVA material (0.012 at 1 MHz). Although the loss of that composite was obviously elevated, it was still at a rather low level (below 0.1, meeting the low-loss demand for high-frequency dielectric materials). In combination with the results in Figure 2a, 40 vol % SiC-filled PVA-based composite possesses the desirable high permittivity (ca. 487) and low loss (0.076) at a high frequency of 1 MHz. More importantly, all of the PVC-based composites were found to have a lower loss than pure PVC material, suggesting a larger potential application advantage compared to the above PVA-based composites. This might be explained by the reduced induced-polarization-triggered interface-leakage loss (compared with the PVA-based system [36]) as well as by a fine linear dielectric characteristic in the PVC material (with rather low remnant polarization and energy storage loss [42]). Note, that all PVC based composites with 10–40 vol % SiC NPs had lower losses than neat PVC, which might be mainly ascribed to an effective dilution effect of NPs onto the PVC matrix in these composites. Due to the relatively weak interaction between SiC and PVC, the introduction of SiC NPs results in a larger free volume for PVC molecules in composites based on the above dilution effect. A lower inner friction force among PVC molecules is obtained in composites, during the dielectric response process, ascribed to the larger free volume. That is to say, it would be easier for electric dipoles (in PVC) to reverse in PVC-based composites than that in pure PVC material, under an applied electric field. Therefore, dielectric losses of all PVC-based composites were lower than neat PVC material, see Figure 2b. However, the case was different in PVA-based composites. Although the dilution effect of NPs still existed in PVA-based composites, a relatively strong interaction between PVA and SiC would restrain the reversal of electric dipoles in the PVA matrix. Thus, the losses of PVA-based composites with 20–40 vol % SiC were much higher than neat PVA material, see Figure 2b. For instance, the loss of 30 vol % SiC-filled PVC-based composite was 0.009, which was ca. 50% of that of neat PVC (0.017) at 1 MHz. As 40 vol % SiC NPs were blended with PVC material, the composite could obtain a very high permittivity (ca. 324) and low loss (0.014) simultaneously, at 1 MHz. To sum up, in order to simultaneously achieve high-k and low-loss properties in composites, a mild induced polarization between filler and matrix would be more helpful to a favorable synergy with electric percolation of the filler than a strong induced polarization.

**Figure 3:**
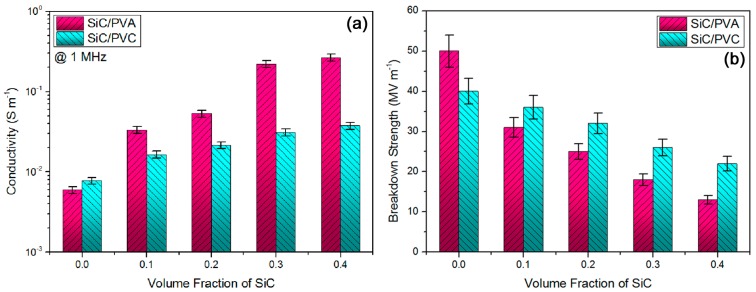
(**a**) Alternative current (ac) conductivity results showed relatively high electric insulation property for PVC-based composite system, and (**b**) breakdown strength data further confirmed advantage of the PVC-based system as high insulation materials.

Usually, a higher electrical conductivity (denoting a larger leakage conduction) would lead to a higher dielectric loss for most of the composite dielectrics. Thus, a low electrical conductivity has been highly desired for high-frequency oriented composite dielectric materials [43]. In Figure 3a, alternative current (ac) conductivity data of two composite systems at 1 MHz are exhibited, as a function of the SiC volume concentration. In general, their ac conductivity varied with a similar trend to their permittivity in Figure 2a, namely, the introduction of SiC could increase the ac conductivity of composites. This could be clarified by a higher inherent conductivity of beta-SiC compared to the two insulating polymer materials. As for the PVA-based composite system, ac conductivity was greatly promoted as the SiC concentration was improved from 20 vol % to 30 vol %. This suggested an electric percolation in the PVA-based system as discussed above. Meanwhile, a strong induced polarization between conducting SiC and ultrahigh polarity PVA might be achieved at an SiC concentration close to 30 vol %, contributing to a high interface leakage-conductance and thus a high ac conductivity. For example, when the SiC concentration was elevated from 20 vol % to 30 vol %, ac conductivity of PVA-based composites was increased by ca. 315% at 1 MHz. Both PVA-based composites bearing 30 vol % and 40 vol % SiC NPs had relatively high ac conductivity data (over 0.1 S·m^−1^) denoting their low insulation disadvantages for high-frequency applications, although high permittivity and low loss could be obtained in them. By contrast, the desired low-level ac conductivity (below 0.04 S·m^−1^) was always maintained in all of the PVC-based composites. With an increase of SiC concentration, ac conductivity of the PVC-based system slowly increased, suggesting a relatively high electric insulation property owing to a mild interface induced polarization between SiC and medium polarity PVC material. The merit of the SiC/PVC composite system was shown again. At a SiC content of 40 vol %, the PVC-based composite exhibited a relatively low ac conductivity of ca. 0.037 S·m^−1^ (about 4.8 times of neat PVC) at 1 MHz.

Apart from the low conductivity of composite dielectrics, their high electric breakdown strength greatly emphasizes their advantages [44]. In Figure 3b, the breakdown strength results of these samples corresponding to their ac conductivity results in Figure 3a were obtained. With an increase of SiC volume fraction, the breakdown strength of both composite systems was found to gradually reduce with an almost linear tendency. Reduction of the breakdown strength should be rooted in the introduction of conductive SiC into the insulating polymer materials. Although PVA had a higher intrinsic breakdown strength compared to PVC, the breakdown strength of the PVA-based system reduced more quickly than that of PVC-based one, with an increase of SiC concentration. This might be caused by a combination of strong induced polarization and electric percolation in the PVA-based composite system. The highly retained breakdown strength for the PVC-based system originated from an optimized synergy between polarization and percolation. For instance, at 30 vol % of the SiC loading concentrations, PVC-based composite had a breakdown strength of ca. 26 MV·m^−1^ (reduced by 35% compared with neat PVC), while the PVA-based counterpart had a breakdown strength of ca. 18 MV·m^−1^ (decreased by 64% compared with pure PVA). Even though a percolation was formed at 30 vol % SiC concentration, the PVC-based composite could possess a favorable high breakdown property. The gap between the breakdown strength data of the two nanocomposite systems may be ascribed to a difference between the induced polarization behaviors of both [36]. To summarize, PVC-based composites showed their advantages once again with respect to their electric breakdown property.

### 3.3. Contribution of Induced Polarization to Conductivity of Nanocomposites

**Figure 4:**
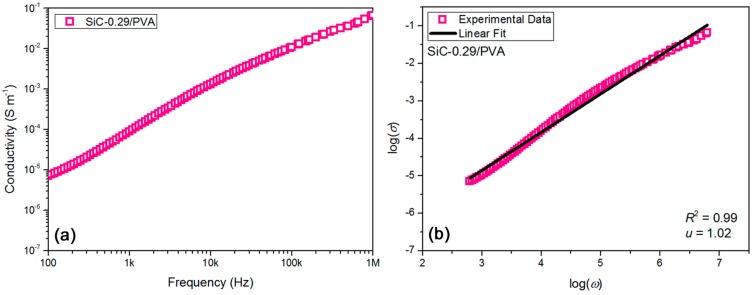
(**a**) AC conductivity result confirmed nice insulation property for SiC-0.29/PVA composite sample, and (**b**) linear fitting of tested data showed elevated *u* value of 1.02 for SiC-0.29/PVA sample and confirmed contribution of induced polarization as ca. 30% to composite conductivity.

In our previous work [45], it was found that an interface induced polarization between the Si-based ceramic filler and the polymer matrix lead to an increase of the conductivity and dielectric constant of the ceramic filler, as well as enhancement of the conductivity and permittivity of composites. In this work, a strategy of combining the induced polarization with electric percolation was raised and executed, as discussed above. Obviously, the high-k property of as-prepared composites was closely connected with the elevated high conductivity of those composites. The high conductivity of composites should come from two aspects, namely the contributions of induced polarization and electric percolation. During past decades, the relation of composite conductivity and filler percolation has been widely researched [46]. However, the specific contribution of induced polarization to an increase of the conductivity of nanocomposites prepared in this work has not been clarified. In Figure 4a, the ac conductivity result of 29 vol % SiC-filled PVA-based composite (abbr. SiC-0.29/PVA) as a function of testing frequency, varying between 100 Hz and 1 MHz, was exhibited. The reason for selecting the SiC-0.29/PVA sample was that 29 vol % should be near the percolation threshold (slightly lower than 30 vol %, discussed above). The ac conductivity was improved in an almost linear trend with an increase of frequency, suggesting a relatively good insulating feature for the sample under a low applied electric field [37]. The ac conductivity changed between 7 × 10^−6^ S·m^−1^ and 0.066 S·m^−1^.

**Figure 5:**
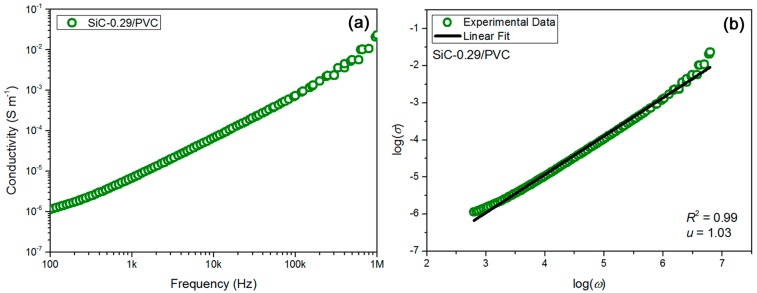
(**a**) AC conductivity result proved the high insulation property for SiC-0.29/PVC sample, and (**b**) linear fitting of measured data showed an elevated *u* value of 1.03 for this sample and also confirmed contribution of induced polarization as ca. 30%.

In classic electric percolation theory, both Ohmic and non-Ohmic conduction mechanisms in composite dielectrics have been proposed [47]. The former is induced after percolation, while the latter is triggered before percolation. As the volume concentration of conducting filler is near the percolation threshold, the ac conductivity (*σ*) of composite has much to do with the angular frequency (*ω*) of the applied ac electric field (*ω* = 2π*f*, *f* is measuring frequency). The relation has been expressed as ‘*σ* ∝ *ω^u^*’ (*u* is the critical exponent) [48]. In Figure 4b, the relation between log(*σ*) and log(*ω*) for the SiC-0.29/PVA sample was obtained, based on the known data in Figure 4a. When a fine linear fitting (adjusted *R*^2^ of the fitting was 0.99) was made onto the data in Figure 4b, the *u* value could be achieved as 1.02 for the sample. As expected, this *u* value (1.02) was much higher than the universal *u* value (0.70) [48] predicted by percolation theory. The gap between the two *u* values was calculated to be 0.32, which may be attributed to the contribution of induced polarization to the ac conductivity of this sample. Therefore, the ac conductivity of the SiC-0.29/PVA sample could be deemed to come from two parts, namely ca. 31% from interface induced polarization and 69% from classic electric percolation. To further prove this deduction, 29 vol % SiC-filled PVC-based composite sample (abbr. SiC-0.29/PVC) was investigated through a similar process performed for the SiC-0.29/PVA sample above, and the corresponding results for the SiC-0.29/PVC sample were exhibited in Figure 5. Figure 5a showed ac conductivity result of the sample as a function of testing frequency, and Figure 5b verified ‘*u* = 1.03’ at *R*^2^ = 0.99 for the SiC-0.29/PVC sample. Surprisingly, the *u* values were very close to 1.00 in the present two cases. To sum up, the contribution of induced polarization to the conductivity of composites should be ca. 30%. The high-k property of as-prepared nanocomposites originates from a synergy of induced polarization (~30%) and electric percolation (~70%).

### 3.4. Low Dependence of Electrical Properties on High Frequency for PVC Composite

**Figure 6:**
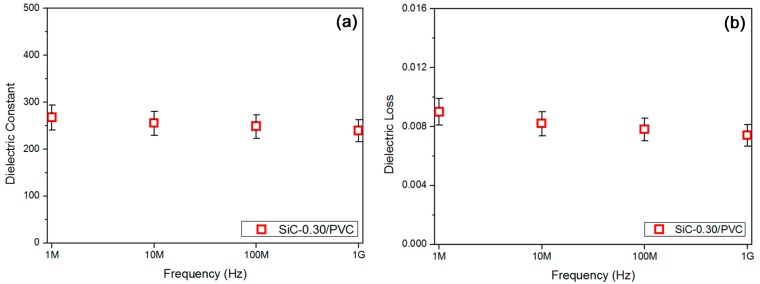
(**a**) Permittivity results showed low dependence of permittivity of SiC-0.30/PVC composite on high frequency, and (**b**) dielectric loss data verified low dependence of loss of that composite on high frequency.

Recently, a low dependence of dielectric and conductive properties on the measuring frequency for high-frequency oriented dielectric materials has been emphasized and explored [49]. Herein, a 30 vol % SiC-filled PVC-based composite sample (SiC-0.30/PVC) was chosen to study the frequency dependence of its electrical performances, aiming at a high-frequency range (1 MHz–1 GHz). The reason for choosing the 30 vol % SiC concentration was to fully utilize electric percolation, and the reason for selecting the PVC matrix was for achieving a mild induced polarization. In Figure 6a, four specific frequencies (1 MHz, 10 MHz, 100 MHz, and 1 GHz) were studied, and a slight reduction for the dielectric constant of the PVC-based composites with an elevation of measuring frequency was found. The permittivity at 1 GHz (ca. 239) was found to reduce by only 10%, compared with that at 1 MHz (ca. 267). Thus, a rather low frequency dependence could be confirmed for the dielectric constant of the SiC-0.30/PVC sample, while the frequencies varied between 1 MHz and 1 GHz. The permittivity of ca. 239 at 1 GHz showed a promising high-frequency application for this PVC-based composite. In addition, the corresponding dielectric loss results for this sample were displayed in Figure 6b. In a similar way, a slight decrease in the loss results was observed, as the frequency was increased. The loss at 1 GHz (ca. 0.0074) was determined to reduce by 18%, in comparison to that at 1 MHz (ca. 0.0090). A relatively low frequency dependence for the loss of this sample could also be verified for a wide frequency range (1 MHz–1 GHz). In most cases, a low loss was always pursued for high-frequency applications of dielectric materials [50]. That rather low loss (~0.0074) at a very high frequency of 1 GHz suggests an energy-efficient merit for the SiC-0.30/PVC sample.

**Figure 7:**
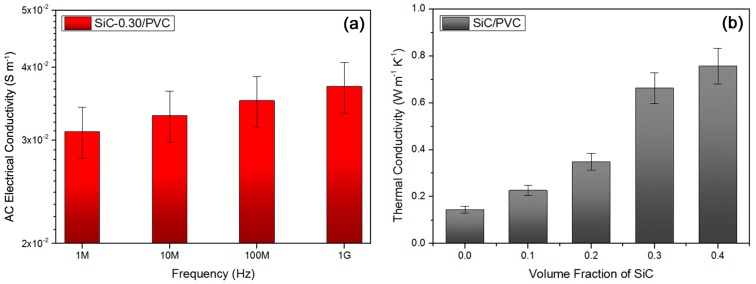
(**a**) AC conductivity data showed relatively low dependence of conductivity of SiC-0.30/PVC composite on high frequency, and (**b**) room-temperature thermal conductivity results suggested formation of heat conductance network and high thermal conductivity for the SiC/PVC composite system.

In addition to the dielectric properties, the frequency dependence for the ac conductive property of the SiC-0.30/PVC sample was also surveyed as shown in Figure 7a. An increase of measuring frequency from 1 MHz to 1 GHz gives rise to a slight increase of the ac conductivity in the sample. When the frequency was improved from 1 MHz to 1 GHz, ac conductivity of the sample was found to increase by 19% (from 0.031 S·m^−1^ to 0.037 S·m^−1^). This could indicate a relatively low frequency dependence for electrical conductivity of this sample in a wide high-frequency scope. Presently, a high thermal conductivity is desired in high-frequency oriented dielectrics materials, leading to a reduction in the possibility of heat-induced electric breakdown [51]. The relation between room-temperature thermal conductivity and SiC volume concentration for the superior SiC/PVC composite system was obtained, as exhibited in Figure 7b. An increase of the SiC loading concentration results in an elevation of thermal conductivity for the samples, ascribed to a far higher inherent heat conductivity of SiC (ca. 83 W·m^−1^·K^−1^) than that of PVC (ca. 0.14 W·m^−1^·K^−1^) [52,53]. As the SiC concentration was improved from 20 vol % to 30 vol %, thermal conductivity of the composites was sharply enhanced (from 0.35 W·m^−1^·K^−1^ to 0.66 W·m^−1^·K^−1^, increased by 89%). This might be illustrated by the formation of a huge thermal conductance network across the composite near the percolation threshold (rather close to 30 vol % too) of SiC NPs with a high heat conductivity [54]. Usually, high thermal conductivity of materials is closely connected with their high electrical conductivity, explaining the almost same percolation threshold (near 30 vol %) for both electric conductance and thermal conductance in present SiC/PVC composites. The highest heat conductivity for the SiC/PVC system was achieved as 0.76 W·m^−1^·K^−1^ (promoted by ca. 443%, in comparison to neat PVC material), at 40 vol % SiC concentration. Therefore, a desired relatively high thermal conductance property could be achieved in the PVC-based composite system.

### 3.5. Hypothetical Synergy Effect between Induced Polarization and Electric Percolation

During past decades, the high-k nanocomposite dielectrics based on electrical percolation of conductive nanofillers have gained great success both in practice and in theory [22]. Plenty of the investigations have found a rather high permittivity in these composites at a low testing frequency (such as 100 Hz) rather than at a high frequency (such as 1 MHz) [55]. Therefore, achieving the desired high-k and low dielectric loss (low *tanδ*) properties in composite dielectrics at high frequencies (1 MHz and even 1 GHz) remains a huge challenge for the researchers. In order to solve this problem, in this work, a facile strategy of combining the traditional electric percolation and new induced polarization (the latter was raised by us [56]) has been proposed. Based on an optimized synergy between electric percolation and mild induced polarization (formed at the SiC/PVC interface), several desired properties discussed above (such as low dielectric loss, high breakdown strength, and high permittivity) have been successfully achieved in as-prepared SiC/PVC nanocomposites.

**Figure 8:**
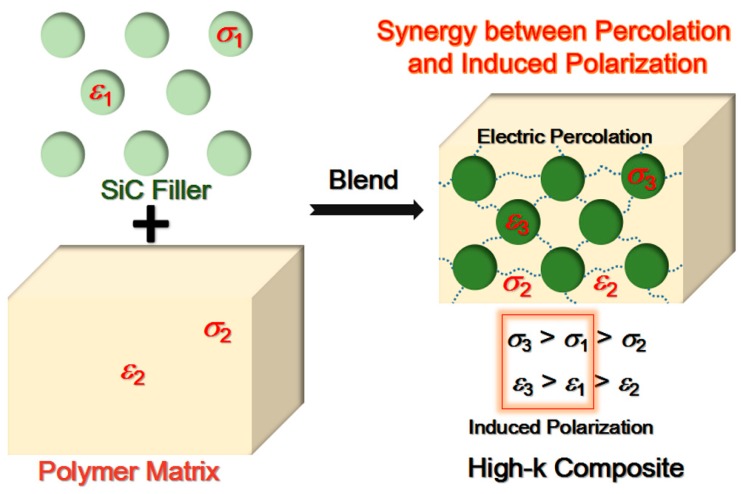
Schematic diagram for physical blend of SiC and polymer showed hypothetical synergy effect between electric percolation and interface induced polarization, contributing to high-k property achieved in the present nanocomposite systems.

In Figure 8, a hypothetical synergy effect between induced polarization and percolation (based on a physical blend of SiC and polymer) was exhibited, contributing to the high-k feature obtained in as-prepared composites. Neat SiC NPs were defined to have a permittivity (*ε*_1_) and conductivity (*σ*_1_), and pure polymer material possessed a permittivity (*ε*_2_) and conductivity (*σ*_2_). Note that *σ*_1_ >*σ*_2_ and *ε*_1_ > *ε*_2_, due to the conductive nature of SiC and insulating nature of the polymer. Once SiC and polymer were physically blended to form the composite, SiC NPs (scattered in the polymer matrix) had a new permittivity (*ε*_3_) and conductivity (*σ*_3_) while the polymer material (as the matrix of composite) still had an unchanged permittivity (*ε*_2_) and conductivity (*σ*_2_). This could be clarified by a significant increase of the overall polarity for SiC NPs and no great change of overall polarity for the polymer, during the interface induced polarization process [45]. Note that *σ*_3_ > *σ*_1_ and *ε*_3_ > *ε*_1_, ascribed to an interface induced polarization between SiC and polymer.

When SiC volume concentration was increased (below the percolation threshold), the total induced polarization in the composite was gradually enhanced [41]. This contributes to an elevation of the permittivity of the entire composite. More importantly, the promoted high conductivity of SiC NPs (compared with the original conductivity of neat SiC NPs, originating from SiC/polymer interface induced polarization) leads to an easier electric percolation (a lower percolation threshold) for SiC NPs in the entire composite. In other words, induced polarization could facilitate electric percolation. With regard to the contribution to an increase of permittivity of the composite, this kind of induced-polarization-assisted electric percolation would be superior to the single electric percolation, we believe. Note that a sufficiently high volume fraction for the SiC/polymer interface phase (introducing more SiC NPs below some critical concentration) should be necessary for effectively forming a favorable induced polarization and forcefully assisting the electric percolation mentioned above. Near the percolation threshold (3D conductive networks formed across the entire composite, see Figure 8), the composite would obtain a more promising high-k property owing to a synergy effect between electric percolation and induced polarization. At the percolation threshold, the composite would be changed from a dielectric to a conductor. In this case, the conduction mechanism in the entire composite would be altered, namely from a non-Ohmic conduction mechanism to an Ohmic conduction one. When the SiC concentration is lower than the percolation threshold, the conduction is induced based on the barrier-tunneling effect (occurs between the conductive SiC NPs across a thin layer of polymer matrix). Herein, it belongs to the non-Ohmic conduction mechanism and the composite shows a dielectric nature. Once the SiC concentration reaches the percolation threshold, all of the SiC NPs would be in direct contact with each other, exactly. Herein, the Ohmic conduction mechanism completely replaces the non-Ohmic one, and the composite exhibits a conductive nature rather than a dielectric nature. The induced polarization-assisted electric percolation proposed in this work, results in a stronger non-Ohmic conduction behavior and higher permittivity in the composite than that for the single electric percolation. In conclusion, high comprehensive electrical properties (including high permittivity, low loss, low electric conductivity, and high breakdown strength) along with high thermal conductivity have been achieved in the SiC/PVC nanocomposite system, based on an optimized synergy effect between mild induced polarization and induced-polarization-assisted electric percolation. This composite system could provide several advantages in the field of high-frequency composite dielectrics materials.

## 4. Conclusions

In this work, a facile and valid strategy for preparing polymer-based nanocomposites with enhanced high-frequency dielectric properties as well as high breakdown property has been presented. Instead of traditional electric percolation for conductive nanofillers, a combination of electric percolation and interface induced polarization has been conducted to aim for a more enhanced high-k property of composites under high frequency. Two nanocomposite systems, namely beta-SiC/PVA and beta-SiC/PVC nanocomposite systems, were fabricated and compared. Based on experiments, PVA-based composites showed an advantage in their high-k property in comparison to PVC-based ones, ascribed to a stronger induced polarization in the former compared to the latter. However, PVC-based composites also had a favorable high-k property. As for the dielectric loss, PVC-based composites were found to be superior to PVA-based ones, attributed to a low interface leakage loss in PVC-based composites and linear dielectric nature in PVC. A low ac conductivity and high electric breakdown strength were found in PVC-based composites, compared with that in PVA-based ones, ascribed to a mild induced polarization between PVC and SiC. Linear fitting of experimental data of the relationship between conductivity and frequency could suggest the sources of conductivity in the composites, namely induced polarization (30%) and percolation (70%). A low dependence of dielectric and conductive properties on high frequency was verified and high thermal conductivity was obtained for PVC composites. An optimized synergy effect between mild induced polarization and induced-polarization-assisted percolation might be responsible for the high comprehensive properties achieved in PVC-based composites. This work may open the way for large-scale fabrication of promising high-frequency composite dielectric materials.
